# Thermodynamics at Very Long Time and Space Scales

**DOI:** 10.3390/e22101090

**Published:** 2020-09-28

**Authors:** Bjarne Andresen, Christopher Essex

**Affiliations:** 1 Niels Bohr Institute, University of Copenhagen, Blegdamsvej 17, DK-2100 Copenhagen Ø, Denmark; 2 Department of Applied Mathematics, The University of Western Ontario, London, ON N6A 5B7, Canada; essex@uwo.ca

**Keywords:** very long timescales, slow time, ideal gas law, new and modified variables

## Abstract

Any observation, and hence concept, is limited by the time and length scale of the observer and his instruments. Originally, we lived on a timescale of minutes and a length scale of meters, give or take an order of magnitude or two. Therefore, we devloped laboratory sized concepts, like volume, pressure, and temperature of continuous media. The past 150 years we managed to observe on the molecular scale and similarly nanoseconds timescale, leading to atomic physics that requires new concepts. In this paper, we are moving in the opposite direction, to extremely large time and length scales. We call this regime “slow time”. Here, we explore which laboratory concepts still apply in slow time and which new ones may emerge. E.g., we find that temperature no longer exists and that a new component of entropy emerges from long time averaging of other quantities. Just as finite-time thermodynamics developed from the small additional constraint of a finite process duration, here we add a small new condition, the very long timescale that results in a loss of temporal resolution, and again look for new structure.

## 1. Introduction

Any observer perceives effects and structure only within a limited time window within which it is able to achieve time resolution and thus establishing ‘before and after’ and causality. At shorter times, we cannot resolve that, at longer times we do not observe any change. We have always experienced laboratory/human scales directly. In more recent times, through inference and some observations, we have extended our time window to shorter timescales, to molecular behavior. This means moving downward by a factor of about 12 or more orders of magnitude. In the slow-time project, we are trying to look the other way, to very long timescales by similarly roughly 12 or more orders of magnitude, in order to search for structures that are invisible on the timescales we experience routinely.

Statistical mechanical modelling of e.g., spin glasses has taken a first step in that direction and found new phenomena, called glassy dynamics, e.g., polynomial time evolution, memory, and recurrence, but still within the laboratory scale universe of variables. An excellent review may be found in [1] and references therein. These phenomena often extend over several decades of time and they involve logarithmic laws, but eventually the usual exponential decay toward equilibrium sets in. These behaviors all involve metastability, i.e., they appear stable for long periods of time. They are also found outside thermodynamics, e.g., describing the decline in extinction rates and scale invariance in the fossil record and the magnetic creep-rate of type-II superconductors [2,3]. In the present study, we go many orders of magnitude beyond those studies and seek possibly new concepts and variables. Does thermodynamics as we know it exist at those time and corresponding length scales?

Our approach is an extension of standard thermodynamics into a new realm very much like finite-time thermodynamics (FTT) was at its conception. FTT added one small condition, a limit on the duration of the process considered. Similarly, slow-time thermodynamics adds the extrapolation to very long times where laboratory dynamics disappears into fluctuations—not the usual equilibrium limit.

The traditional thermodynamic limit in which the system is assumed in equilibrium at uniform intensive parameters (e.g., temperature) throughout no longer exists. The laboratory variables fluctuate faster than what is observable in slow time, and position has similarly been coarsegrained. The system never reaches even quasi-equilibrium in our usual variables. However, perhaps some other variables do?

The aim is to treat directly the effects of long timescales on physics or chemistry as time resolution is lost. The distinct regimes of physics, such as the atomic and laboratory regimes, are familiar. There is a clear hierarchy which has the distinctive property that each regime can “ignore” underlying ones, even though they must be in fundamental agreement. That property is known as closure [4], following the terminology arising from the historical problem of turbulence. Closure emerges through a process that induces new relationships between existing or modified quantities in some limit, yielding a system of equations that can be solved without reference to the underlying regime.

The central question the slow-time approach asks is whether a new regime or new regimes emerge on long timescales and since equilibration expands at a certain rate, correspondingly coarsened space scales. Loosely speaking that question puts an observer in a situation not unlike trying to view the laboratory regime from atomic or kinetic scales. From the standpoint of such scales, the laboratory regime induces new physical variables such as temperature, while burying specific dynamical variables in the loss of resolution whereby entropy emerges.

**Figure 1:**
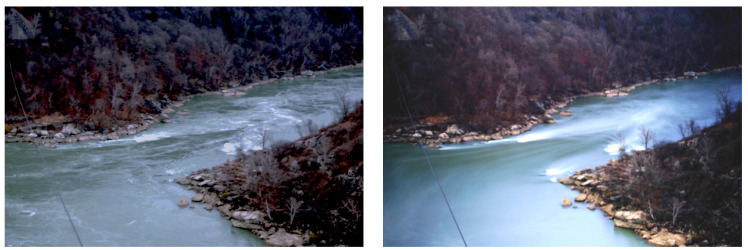
Two images of the same Niagara Falls downstream flow. The left image is an exposure of 1/2 sec, while the right hand image is exposed for 50 sec. Note the flow features visible in the right hand image (stream lines, bow waves, standing waves, vortices, etc.) that are not clearly visible or simply invisible in the left image due to the “noise” of local fluctuations.

**Figure 2:**
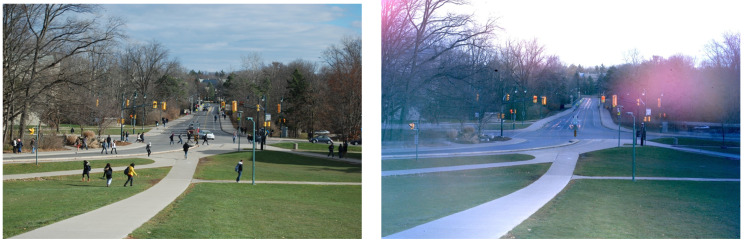
A busy intersection with lots of students, cars, trucks, and busses moving about. The left frame is a normal picture taken at 1/100 sec, the right frame is exposed for 10 min. No moving objects are seen anymore, except for a few very faint shadows. The multiple cars in the left turning lane sit waiting for green, leading to multiple images of running lights in that position. The red-yellow-green traffic lights are all lit at the same time, on average.

Our first experiments consisted of flowing water, in this case the Niagara River just below the falls. The left picture of Figure 1 has a 1/2 s exposure. The right picture is exactly the same but with a strong filter allowing a 50 s exposure. The unsteady flow disappears in favor of smooth streamlines turning to the right and vivid standing and bow waves previously invisible in the “noise” of local fluctuations. We see similar “tranquil” situations in slow-time pictures of trees in the wind and of busy traffic in Figure 2. These pictures illustrate the presence of structure appropriate to different timescales.

## 2. The Slow-Time Probability Density Function

In our first exploration of a fluctuating system at long times [5], we let temperature and flow velocity fluctuate in a simple homogenous system to produce probability density functions (PDF’s) appropriate for long timescales. It identified key features of PDF’s that must arise without consideration of the small-scale local equilibrium systems per se.

The standard thermal Gaussian molecular velocity distribution that is centered around *u* is

(1) $$\begin{array}{c} p_{u}\left(v\right)={\left(\frac{m}{2kT}\right)}^{1/2}\frac{1}{\sqrt{\pi }}\;e^{-\frac{m}{2kT}{\left(v-u\right)}^{2}}, \end{array}$$

where *m* is the particle mass, *k* the Boltzmann constant, and *T* the standard temperature. Fluctuating the rest velocity *u* (wind) about zero with a given variance $\sigma_{u}$ results in a new Gaussian distribution [5],

(2) $$\begin{array}{c} p_{\theta }\left(v\right)={\left(\frac{m}{2k\theta }\right)}^{1/2}\frac{1}{\sqrt{\pi }}\;e^{-\frac{m}{2k\theta }v^{2}} \end{array}$$

but with a modified temperature θ,

(3) $$\begin{array}{c} \theta =T+\frac{\sigma_{u}^{2}m}{k} \end{array}$$

that embeds the fluctuations of the wind velocity *u* in the form of its variance. In other words, the wind has been thermalized. However the particle velocity *v* has not been. *v* persists as a valid quantity on long timescales. Similar quantities that carry over to slow time are particle number, energy, and under certain conditions volume. The fluctuations in wind velocity have become thermalized just like the molecular velocities are on the laboratory timescale, a sort of mega Brownian motion. Because the expression (2) is still a standard thermal Gaussian distribution, just with a new temperature variable in the exponent, θ is indeed a real temperature for its regime, not some sort of “noise temperature”. The wind has simply been thermalized. Fluctuating wind on the laboratory scale is no different from fluctuating molecular velocities on the atomic scale. We could, of course, equally well have fluctuated the reference velocity *u* around a non-zero value; that would not have changed the conclusions above. A realistic wind fluctuation in our daily weather is $\sigma_{u}∼5\;m/s$, which makes the correction term $\sigma_{u}^{2}m/k∼0.1\;K$, a change that is negligible under normal conditions. θ is not a generalization of temperature, it is an emerging feature of long-time fluctuations of wind and will, therefore, in all places replace the laboratory scale temperature *T*.

Fluctuating temperature, on the other hand, is more delicate, because it does not only appear in the exponent of the molecular velocity distribution (1), but also in the prefactor. That leads to the very interesting normalized functional form [5]

(4) $$\begin{array}{c} p_{uT}\left(v\right)=\int_{-\infty }^{\infty }p_{u}p_{\xi }d\xi =\frac{w^{3}\psi_{0}}{\sqrt{\pi }{\left(v^{2}+w^{2}\right)}^{3/2}}\;e^{-\frac{w^{2}\psi_{0}^{2}v^{2}}{v^{2}+w^{2}}}, \end{array}$$

valid for $w\psi_{0}≳2$. In this equation, *v* is the particle velocity and ψ(θ) is the precision of the Gaussian velocity distribution (1), which is itself fluctuating around the central value

(5) $$\begin{array}{c} \psi_{0}\equiv \psi \left(\theta \right){|}_{\theta =\theta_{0}}=\sqrt{m/2k\theta_{0}} \end{array}$$

according to the Gaussian distribution

(6) $$\begin{array}{c} p_{\xi }=\frac{w}{\sqrt{\pi }}\;e^{-w^{2}\xi^{2}} \end{array}$$

where ξ is the fluctuating part of ψ, $\psi =\psi_{0}+\xi$ and with θ defined in (3). Thus, *w* is the Gaussian precision of the fluctuations in ψ with units of velocity. For mathematical convenience, we work with the Gaussian precision instead of the standard deviation. For standard deviation σ the precision ψ=1/σ. Thus a larger precision means a tighter distribution. We could, of course, have chosen another type of fluctuation for ψ than the Gaussian (6) as long as it approaches zero at large argument values sufficiently rapidly to be normalizable. The results would have been qualitatively the same, the mathematics just more complicated.

**Figure 3:**
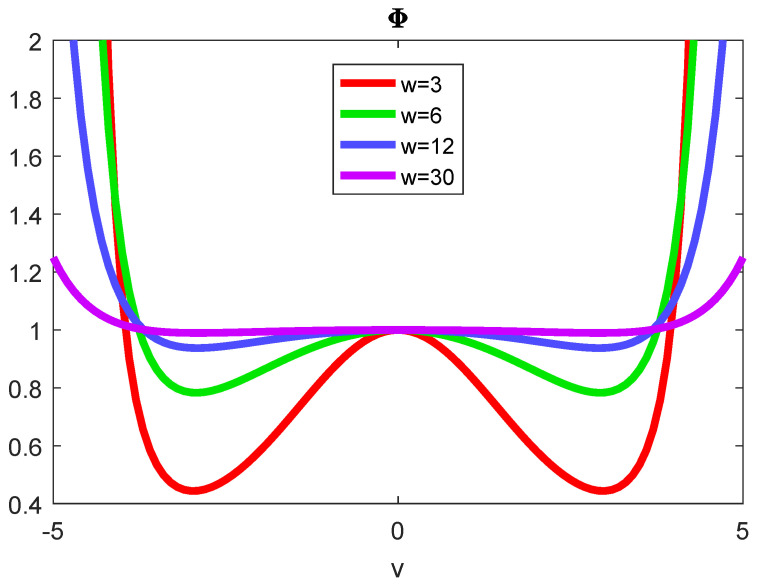
The function $\Phi =p_{uT}/p_{u}$ relating the PDF for fluctuating velocity as well as temperature for four different precisions of precisions *w* to the PDF with an exact precision of temperature. The two distributions are identical, i.e., Φ=1, for v≪w.

$p_{uT}\left(v\right)$, Equation (4), is a very interesting function in that it is a conventional Gaussian for large *w* (i.e., very narrow fluctuation of ψ), but becomes a power function for small *w*. That means that (4) is not a proper thermal distribution, in other words, the concept of temperature does not extend to long timescales where also the precision of the velocity distribution fluctuates. Besides formally being the precision of the precision of the *v* fluctuations, *w* is the transition velocity above which the effects of temperature fluctuations become unthermalizable due to the heavy tails. Figure 3 showing the function $\Phi =p_{uT}/p_{u}$, i.e., the PDF for fluctuating temperature (4) as compared to the Gaussian PDF for fluctuating wind (1), illustrates this. As long as v≪w, the value is 1, but, outside that range, considerable deviations appear.

So far, we have been considering a one-dimensional system which fluctuated as a whole. Especially for very long time and spatial scales we need to be more realistic and allow for local fluctuations around local equilibria. Such local equilibria are widespread in physics from the smallest to the largest scales. Those systems do not even need to be in steady state. We define this situation as *fluctuating local equilibrium* (FLE). It implies the existence of fluctuating scalar and vector fields throughout the system, each local pocket will fluctuate by itself. We use these properties to determine a slow-time relationship between moments in an FLE system that does not employ usual variables in the laboratory regime valid for finite *w* in order to arrive at a long timescale equation for an ideal gas in an FLE system. In addition, we assume spatial isotropy for mathematical convenience.

## 3. The Slow Time Ideal Gas Law

Because the physical world is not continuous to the extreme but eventually discrete, all moments of $p_{uT}\left(v\right)$ (4) are convergent for sufficiently large *w*. Let $M_{n}\left\{g\left(v\right)\right\}\equiv \int_{D}v^{n}g\left(v\right)\;dv$ be the $n^{\mathrm{th}}$ moment of g(v). Subsequently, $M_{0}\left\{p_{uT}\left(v\right)\right\}=1$, and $M_{1}\left\{p_{uT}\left(v\right)\right\}=0$ while the second moments add up to the energy *E* involving all components of v,

(7) $$\begin{array}{c} E=\frac{mN}{2}\sum_{i}M_{2}\left\{p_{uT}\left(v_{i}\right)\right\}=\frac{mN}{2}\sum_{i}M_{2}\left\{p_{u}\left(v_{i}\right)\right\}+\frac{mN}{2}\sum_{i}M_{2}\left\{p_{uT}\left(v_{i}\right)-p_{u}\left(v_{i}\right)\right\}, \end{array}$$

where *N* is the number of particles in the entire FLE system and the summations over *i* are the three dimensions of physical space, x,y,z. For some suitable function $f(w,v_{*})$, this may be written as

(8) $$\begin{array}{c} E=\frac{3}{2}Nk\theta +Nf\left(w,v_{*}\right). \end{array}$$

The first term is the standard energy expression for the thermal distribution (1), i.e., the first term on the right hand side of (7), while $f(w,v_{*})$ represents the second right hand term. Those are the effects due to the thermal fluctuations that depend on their precision of precision *w* and a parameter $v_{*}$ indicating the largest velocity for which a continuous PDF is physically meaningful. I.e., realistically, no particle in the system has a velocity $v>v_{*}$ even though the continuous PDF does have a tiny but non-vanishing value all the way to infinity. This observation of discreteness in Nature allows us to truncate the normalization *v* integral of $p_{uT}\left(v\right)$ from *∞* to $v_{*}$ and thus ensure normalization of the PDF (4) in all situations. The properties of $f(w,v_{*})$ require *w* and $v_{*}$ to be fully determined in terms of the specific structures in play.

The correspondence principle for the FLE systems requires that the energy equation reduces to the classical equation $E=\frac{3}{2}NkT$ in the absence of fluctuations. Clearly, θ→T in that case, but we must also have $\lim_{w\to \infty }f\left(w,v_{*}\right)=0.$ This follows if $\lim_{w\to \infty }p_{uT}\left(v\right)=p_{u}$. To ensure these results, we must either ignore $v_{*}$ or require $w\ll v_{*}$. This allows for us to introduce an expansion at infinity of the form $f\left(w,v_{*}\right)=w^{-2}h+O\left(w^{-4}\right)$, or

(9) $$\begin{array}{c} E=\frac{3}{2}Nk\theta +Nw^{-2}h+O\left(w^{-4}\right), \end{array}$$

where *h* is a constant to be determined. We do not at present know this slow-time behavior and, thus, must expect h≠0. Its value will depend on specifics unavailable to us a priori with our laboratory scale knowledge, but it is, in principle, something measurable with appropriate instruments from the [deleted the extra ‘the’] slow-time regime.

Discarding higher order terms in (9),

(10) $$\begin{array}{c} E=\frac{3}{2}Nk\theta +Nw^{-2}h \end{array}$$

becomes the slow-time version of the *ideal gas law*, where θ, *w*, and *h* are the natural slow-time regime variables.

## 4. Discussion and Conclusions

To summarize, the slow-time temperature θ reduces to *T* in the no fluctuation limit, but the classical temperature *T* itself no longer exists. The transition velocity, *w*, the statistical precision of fluctuations in the temperature variable, ψ, (5), represents the transition from Gaussian to heavy tail (polynomial) behavior. *h* is the residual at infinity of the correction term in the slow-time ideal gas law (10).

Hoping to define an appropriate slow-time temperature, distinct from θ, analogously to the laboratory regime requires that we have a definite entropy of the FLE in order to be able to calculate a derivative that is analogous to ∂E/∂S. However, there is no fundamental reason to carry over the notion of intensities that are generated from partial derivatives of a function of extensities. Like classical temperature, this analogy may prove to be unsuitable for the slow time regime.

Entropy does not exist at the atomic scale, since all motion in principle can be monitored and, thus, is represented as kinetic energy. On the laboratory scale that small random motion is coarse grained away (blurred), but its average effect remains in the form of entropy. In slow time, an analogous effect turns randomly variable winds into a temperature contribution to the new thermalized wind temperature θ.

However, *h* (9) is different, because it describes the large-scale behavior of the system. The integral *h* should have a measurable value, but that value is not knowable from classical theoretical principles on the laboratory scale. We still lack an expression that is analogous to $S=-k\sum_{i}p_{i}\ln p_{i}$. The Maxwellian has unsuitable tail behavior to address this matter. The new term in the slow-time ideal gas law (10), *h*, captures features that are invisible on shorter timescales, e.g., the laboratory scale. In principle, *h* is a new observable of the slow-time regime. We call it *epitropy*.

Similar slow-time behavior, not seen on shorter timescales, may be found in simple numerical simulations. Consider the Rösler equations,

(11) $$\begin{array}{cc} \dot{x} & =-y-z \end{array}$$

(12) $$\begin{array}{cc} \dot{y} & =x+Ay \end{array}$$

(13) $$\begin{array}{cc} \dot{z} & =xz-Cz+B. \end{array}$$

**Figure 4:**
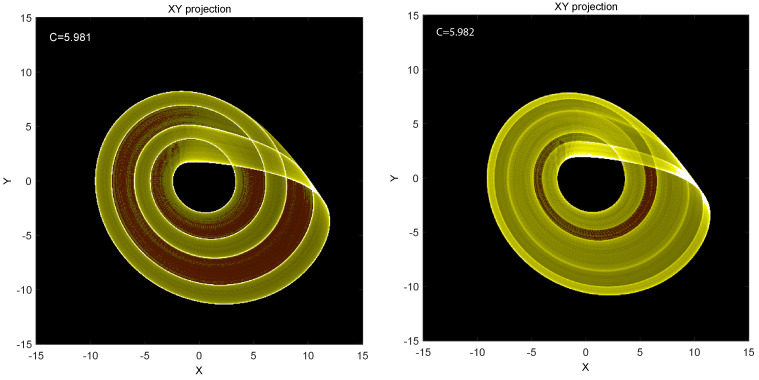
XY-projections of a $10^{7}$ step iteration of the Rösler equations with the parameters A=B=0.2 and C=5.981 (left) and C=5.982 (right). We see that: (i) the many trajectories have a clear large-scale structure with bands of dense population (yellow) and bands of minimal population (brown) very clearly separated although adjacent. (ii) The tiny difference in the C parameter, from 5.981 to 5.982, dramatically changes the picture.

They describe a chaotic three-dimensional system, where the long-time behavior cannot be observed through a short calculation. Figure 4 shows the XY projection of a long-time calculation ($10^{7}$ steps) with the parameters A=B=0.2 and C=5.981 (left) and C=5.982 (right). We can make two observations from these pictures: (i) the many trajectories have a clear large-scale structure with bands of dense population (yellow) and bands of minimal population (brown) very clearly separated although adjacent. Neither the equations nor a few individual trajectories indicate such a behavior. (ii) The tiny difference in the C parameter, from 5.981 to 5.982, dramatically changes the picture. This is slow-time behavior, not seen on cursory plots.

A strange attractor gives some insight into the slow-time picture. Figure 4 shows two instances of the attractor with slightly different parameter settings. Someone computing the trajectory over short time sees trajectories and not densities. Outside of a bifurcation point, they will not detect any qualitative differences in the attractor in the two cases, but, after long time integrations, one notices shifts in the densities of trajectories that only become visible and understandable in the coarsegraining that is implied by densities. One might connect density distributions in space with the system parameters to gain a qualitative understanding of long-time behaviors without revealing any obvious differences to the short-time trajectories. The long-time behaviors are invisible on the short timescales.

Finite-time thermodynamics added the seemingly small additional constraint that the process in question proceed during a finite time. However, it had a profound effect and led to concepts, like maximum power, minimum entropy production, time dependent potentials, optimal paths, and a lot more and spread to a much wider range of applications than usually called thermodynamic. In this paper, we use the same approach by adding a small new condition, a very long timescale, and again look for new structure. We do not claim to have built a full new theory of slow time, only defined some new concepts, and found surprising observations for particular functional dependencies, like the Gaussian fluctuations laws (6). Other explicit PDF’s would have resulted in somewhat different behavior, but the new effects, like a modified temperature (3), a non-Gaussian long-time behavior (4), and the appearance of epitropy (9) would not have been affected. So far, we have only scratched the surface, there is much more to come. The derivation of an "ideal gas law" and a new contribution (epitropy) to the entropy in slow-time are encouraging.

## References

[1] Sibani P., Boettcher S., Jensen H.J. (2020). Record dynamics of evolving metastable systems: Theory and applications. arXiv.

[2] Newman M.E.J., Eble G.J. (1999). Decline in extinction rates and scale invariance in the fossil record. Paleobiology.

[3] Oliveira L.P., Jensen H.J., Nicodemi M., Sibani P. (2005). Record dynamics and the observed temperature plateau in the magnetic creep-rate of type-II superconductors. Phys. Rev. B.

[4] Essex C. (2011). Climate theory versus a theory for climate. Int. J. Bifurcation Chaos.

[5] Essex C., Andresen B. (2015). Maxwellian velocity distributions in slow time. J. Non-Equil. Thermod..

